# Sublingual Buprenorphine and Methadone Maintenance Treatment: A Three-Year Follow-Up of Quality of Life Assessment

**DOI:** 10.1100/tsw.2005.52

**Published:** 2005-05-24

**Authors:** Salvatore M. Giacomuzzi, Markus Ertl, Georg Kemmler, Yyvonne Riemer, Alexander Vigl

**Affiliations:** University Department of Psychiatry, Anichstraße 35, 6020 Innsbruck, Austria

**Keywords:** oral methadone, sublingual buprenorphine, quality of life, QOL, maintenance treatment, addiction, drug abuse, Austria

## Abstract

This study was conducted to compare long-term outcome effects on the quality of life (QOL) of oral methadone with sublingual buprenorphine maintenance treatment. The QOL status of opioid-dependent patients was assessed using the German version (“Berlin Quality of Life Profile”) of the Lancashire Quality of Life Profile. Physical symptoms were measured using the Opiate Withdrawal Scale (OWS). Urine tests were carried out randomly to detect additional consumption. In the first study period, 53 opioid-dependent subjects were enrolled and 25 could be reached after 3 years. The retention rate was 50% for methadone and 45% for buprenorphine (*p* = 0.786). Baseline values of the total sample (completers and noncompleters) QOL and somatic complaints did not show significant differences between the two treatment groups. QOL characteristics at 6 months of treatment of the buprenorphine completer and noncompleter groups differed significantly regarding job (*p* = 0.013), family, and total score of physical symptoms (*p* = 0.002), in which the completer group showed the more favorable values. Concerning physical symptoms at 36 months, logistic regression revealed significantly less stomach cramps (*p* = 0.037) and fatigue and tiredness (*p* = 0.034) in buprenorphine compared to the methadone. Moreover, the buprenorphine-maintained group showed significantly less additional consumption of benzodiazepines (*p* = 0.015) compared with methadone participants. It is concluded that opioid addicts improved their QOL and health status when treated with methadone or buprenorphine. In summary, regarding QOL and health status, the present data indicate that buprenorphine is also a useful long-term alternative for maintenance treatment of opioid-dependent patients.

### INTRODUCTION

Quality of life (QOL) is an area of study that has attracted a great deal of interest over the past decade, particularly in the areas of health and social services, but increasingly in medicine and education. QOL

outcomes are being recognized as important prognostic variables that help to predict which patients are most likely to benefit from treatment[1,2,3,4,5,6]. The QOL concept has also been acknowledged as an important tool in the evaluation of drug programs that provides information about functioning and well being that is not captured by traditional measures of substance use, and has begun to play a more prominent role in evaluating the effectiveness of treatment services for substance abusers[7,8,9,10,11, 12,13,14,15,16,17,18,19,20].

Methadone maintenance treatment has been demonstrated to be an effective treatment for opioid addiction and curbs the incidence of HIV[21,22,23,24,25]. Although methadone maintenance treatment has been successful, it is associated with a number of problems[26,27,28,29,30,31]. Therefore, methadone is not the golden standard for all subjects.

In 1996, when buprenorphine treatment was implemented, it was considered an original and promising alternative to methadone treatments[9,32]. The results of Ahmadi anf Ahmadi[33] support the efficacy and safety of oral methadone and sublingual buprenorphine tablets for injection-opioid-dependent patients. The Cochrane Drugs and Alcohol Group meta-analysis[34] highlights the effectiveness of both methadone and buprenorphine treatment on retention in treatment and opiate use. In several studies, buprenorphine was found to be nearly as effective as methadone and a useful alternative for maintenance therapy of opioid-dependent subjects[35,36,37,38,39,40,41,42,43,44,45,46,47,48,49]. Comparative clinical trials have shown comparable levels of effectiveness and safety for both opiate substitution drugs[35,36,37,39,50,51,52,53,54], even if low-dose buprenorphine is not superior to placebo regarding heroin use[34]. However, Barnett et al.[41] concluded that further research is needed to determine if buprenorphine treatment is more effective than methadone in particular settings or in particular subgroups of patients.

This paper is the first to present a follow-up, aimed at comparing oral methadone with sublingual buprenorphine, as measured by QOL, physical symptoms, and additional consumption after a treatment period of 3 years (2001–2004), obtained from patients retained in the maintenance programs.

### METHODS

#### Subjects

Opioid-dependent patients of the first study period (February–April 2001) entered the follow-up. There were 53 opioid-dependent subjects enrolled in the first study period and 25 could be reached after 3 years of maintenance treatment (February–April 2004).

#### Inclusion Criteria

The only requirement for inclusion in the follow-up was participation in the first study period including a confirmed diagnosis of opioid dependence (DSM-IV 304.0) and informed consent.

#### Ethics

As all participants were within a normal methadone or buprenorphine program, there was no need to approve the study by the human subject ethical committee. It was explained that records would be assigned a code number and that specific patient identifiers would not be kept in research code.

### Measures

We assessed the QOL status of opioid-dependent patients admitted to a methadone or buprenorphine maintenance program using the German version ("Berlin Quality of Life Profile") of the Lancashire Quality of Life Profile, a well-established measurement instrument[55,56,57,58,59,60,61]. This instrument has proved to be valid, reproducible, and responsive to changes over a wide range of QOL and health status, as well as appropriate for the evaluation of medical and social interventions as an outcome measure for group comparisons[60,61,62]. Furthermore, a questionnaire was used to register the patients' specific demographic characteristics (e.g., length of heroin use). Physical symptoms were measured using the Opiate Withdrawal Scale (OWS)[63,64,65,66]. Urine screening tests were carried out regularly, but at random time intervals, to detect additional consumption. Urine samples of each client were always temperature tested. Patients with positive urine toxicology results were not retested more often. All urine samples were tested at the Institute of Legal Medicine in Innsbruck.

### Substitution Program

History and physical examination supported the judgment on the part of the physician that the patient was a candidate for methadone or buprenorphine maintenance program and that such treatment was indicated on the basis of a thorough clinical evaluation[67,68,69,70,71,72,73]. The initial dose, therefore, was based on the physician's evaluation of the history and present physical condition of the patient with added knowledge of local conditions, such as the relative purity of the appropriate street drugs. Benzodiazepines are generally not prescribed for these patients at our department.

The methadone and buprenorphine maintenance treatments are designed to reduce illegal and harmful opioid use (e.g., heroin, codeine, dilaudid, oxycodone, retard. morphine) along with the many problems (e.g., crime, disease, death) associated with this addiction. The main outcome of these treatment interventions are to decrease or even eliminate opioid use to help stabilize the individuals so that they will not return to previous substance abuse behavior patterns. Main aims are maintenance rather than abstinence. Abstinence may be a long-term goal. The treatments are multidisciplinary; this means that methadone or buprenorphine maintenance is not the only treatment, but that other needs of the patient (be they medical, social, legal, psychiatric, or something else) are addressed at the same moment. Both of the maintenance programs, therefore, included psychosocial care in addition to medical care.

### Statistical Analysis

Statistical analysis was performed using SPSS, version 11.5 (SPSS Inc.). In view of the moderate sample size and the fact that scales were not normally distributed, nonparametric tests were used throughout the analysis. Differences between the two study groups were analyzed using the $\chi 2$-test for nominal data sets. The Fisher exact test was performed for $2 \times 2$-tables. Group comparisons with respect to ordinal or continuous variables were performed using the Mann-Whitney U test. Furthermore, an analysis of covariance was performed to adjust QOL scores at 36 months for possible effects of baseline QOL scores. A logistic regression was performed to adjust nominal data sets. Within the substitution groups, pre-post comparisons (baseline vs. 36 months) for nominal data were evaluated with the McNemar-test. The Wilcoxon-test was applied for ordinal and interval data sets. All $p$-values reported are two-tailed.

## RESULTS

### Early Termination and Follow-Up

Of the 53 initial participants, 25 (47%) did complete a maintenance period of 3 years. The retention rate was 50% (12 subjects of 24) for methadone and 45% (13 subjects of 29) for buprenorphine-treated subjects ($p$ = 0.786). In the methadone treatment group, a total of 12 patients discontinued treatment for the following reasons: unknown discharge, 4; death, 1 (intoxication); successful detoxification, 2; and change of substitution treatment, 5. In the buprenorphine group, a total of 16 participants did not continue treatment because of death, 2 (intoxication); successful detoxification, 2; unknown discharge, 3; prison, 1; and change of substitution treatment, 8. All of these patients dropped out during the last 2.5 years.

Throughout the last 3 years, all remaining patients showed excellent compliance within the established program. Demographic data for the study participants (completer analysis) are shown in Table 1. Thirteen subjects (7 females and 6 males) receiving sublingual buprenorphine were compared with 12 subjects (3 females and 9 males) treated with racemic methadone. No significant differences between the two treatment groups were seen. At the end of the study period, the mean methadone dose was 55.4 mg (range = 5–120 mg) and the mean buprenorphine dose was 8.4 mg (range = 2–32 mg). Calculated dose conversion for equivalent doses in both programs showed no differences ($p$ = 0.309).

**TABLE 1**
**Demographic Data: Completer Analysis**

| | Methadone (%) | Buprenorphine (%) | *p*-Value |
|---|---|---|---|
| **Employment status** | | | |
| Employed | 50.0 | 53.8 | 1.000 |
| Hours of work per week | 17.7 ± 18.9 | 18.1 ± 19.5 | 0.979 |
| Money per month (€) | 611.7 ± 308.5 | 1013.8 ± 766.1 | 0.247 |
| **Gender** | | | |
| Male | 75.0 | 46.2 | |
| Female | 25.0 | 53.8 | 0.226 |
| **Medical status** | | | |
| Substitution doseage (mg) | 55.4 | 8.1 | 0.113 |
| HIV | 0.0 | 0.0 | — |
| Hepatitis B | 25.0 | 23.1 | 1.000 |
| Hepatitis C | 41.7 | 69.2 | 0.238 |
| **Family status** | | | |
| Married | 33.3 | 30.8 | |
| Single | 66.7 | 61.5 | |
| Widowed | 0.0 | 7.7 | 0.510 |
| Children | 16.7 | 46.2 | 0.202 |
| Age (years) | 29.9 ± 5.5 | 33.4 ± 7.6 | 0.270 |
| Length of addiction (years) | 11.3 ± 3.5 | 11.3 ± 5.1 | 0.728 |

QOL characteristics of the two study groups are detailed in Table 2. According to the self-reported Lancashire Quality of Life Profile, statistically significant differences at baseline were found regarding partnership ($p$ = 0.028) and the overall satisfaction score ($p$ = 0.019), in which the patients of the buprenorphine group showed the more favorable values. Differences between both groups regarding other QOL scores were not significant. The outcome measures for physical symptoms are shown in Table 2, in which the patients of the buprenorphine group at baseline showed significantly more favorable values regarding poor appetite ($p$ = 0.047), feelings of coldness ($p$ = 0.047), and muscular tension ($p$ = 0.047).

**TABLE 2**
**Subjective Scores of the Lancashire Quality of Life Profile and Percentage of Physical Symptoms: Completer Analysis**

| Satisfaction scores of different domains[1] (range 1-7) | Methadone baseline | Buprenorphine baseline | p-value baseline | Methadone at 6 months | Buprenorphine at 6 months | p-value 6 months | Methadone after 3 years | Buprenorphine after 3 years | p-value 3 years | Baseline-adjusted p-values 3 years between Methadone and Buprenorphine | p-value Methadone group between baseline and 3 years | p-value Buprenorphine group between baseline and 3 years |
|---|---|---|---|---|---|---|---|---|---|---|---|---|
| **Job** | 3.6 (1.8)[1] | 5.1 (2.1) | 0.087 | 4.5 (2.0) | 5.6 (1.8) | 0.186 | 4.8 (1.7) | 5.0 (2.2) | 0.728 | 0.944 | 0.179 | 1.000 |
| **Leisure time** | 3.7 (1.8) | 4.8 (1.5) | 0.123 | 4.9 (1.7) | 5.1 (1.3) | 0.894 | 3.7 (1.4) | 5.0 (1.6) | **0.035** | 0.114 | 0.953 | 0.407 |
| **Finances** | 3.1 (1.9) | 3.3 (1.6) | 0.728 | 4.0 (1.9) | 4.2 (2.0) | 0.852 | 2.9 (1.1) | 4.0 (2.0) | 0.137 | 0.082 | 0.766 | 0.150 |
| **Housing** | 4.7 (1.4) | 5.2 (1.3) | 0.347 | 6.0 (1.1) | 5.4 (1.4) | 0.123 | 4.4 (1.5) | 5.7 (1.5) | **0.010** | 0.067 | 0.813 | 0.142 |
| **Law and security** | 5.7 (0.7) | 5.7 (1.4) | 0.437 | 5.7 (0.7) | 6.0 (1.2) | 0.110 | 5.6 (1.0) | 6.3 (1.2) | **0.035** | 0.112 | 0.787 | **0.026** |
| **Family** | 5.5 (1.0) | 5.6 (1.3) | 0.650 | 4.9 (1.7) | 6.0 (1.4) | 0.068 | 5.0 (1.3) | 5.4 (1.9) | 0.247 | 0.555 | 0.321 | 0.968 |
| **Partnership** | 4.3 (1.1) | 5.6 (1.2) | **0.028** | 5.2 (1.9) | 5.8 (1.4) | 0.611 | 4.3 (1.9) | 4.5 (2.3) | 0.894 | 0.113 | 1.000 | 0.058 |
| **Friends and acquaintances** | 5.0 (1.4) | 5.0 (1.5) | 1.000 | 5.7 (1.1) | 5.5 (1.4) | 0.810 | 5.3 (0.7) | 4.9 (1.9) | 0.728 | 0.409 | 0.452 | 0.905 |
| **Physical health** | 3.4 (1.6) | 4.4 (2.0) | 0.205 | 4.3 (1.4) | 4.9 (1.7) | 0.320 | 3.8 (1.3) | 4.5 (1.9) | 0.247 | 0.715 | 0.541 | 0.476 |
| **Psychical health** | 3.8 (1.6) | 5.0 (1.8) | 0.077 | 4.3 (1.7) | 5.2 (1.6) | 0.186 | 3.9 (1.8) | 4.6 (1.8) | 0.320 | 0.817 | 0.877 | 0.521 |
| **Self-esteem[2]** | 6.0 (3.2) | 7.4 (3.1) | 0.247 | 7.0 (3.4) | 8.3 (2.6) | 0.225 | 6.8 (2.0) | 7.8 (3.0) | 0.077 | 0.537 | 0.504 | 0.414 |
| **Overall satisfaction** | 3.1 (1.2) | 4.7 (1.6) | **0.019** | 4.0 (1.5) | 5.4 (1.3) | **0.030** | 3.8 (1.3) | 5.0 (1.8) | **0.030** | 0.278 | 0.102 | 0.418 |
| **Percentage of physical symptoms [%]** | | | | | | | | | | | | |
| **Stomach cramps** | 33.3 | 15.4 | 0.378 | 58.3 | 15.4 | **0.041** | 50.0 | 7.7 | **0.030** | **0.037** | 0.688 | 1.000 |
| **Twitching of muscles** | 66.7 | 46.2 | 0.428 | 33.3 | 23.1 | 0.673 | 33.3 | 38.5 | 1.000 | 0.791 | 0.219 | 1.000 |
| **Fatigue or tiredness** | 58.3 | 53.8 | 1.000 | 58.3 | 7.7 | **0.011** | 75.0 | 30.8 | **0.047** | **0.034** | 0.625 | 0.375 |
| **Runny eyes** | 25.0 | 23.1 | 1.000 | 25.0 | 7.7 | 0.322 | 50.0 | 15.4 | 0.097 | 0.072 | 0.375 | 1.000 |
| **Inability to fall asleep** | 75.0 | 46.2 | 0.226 | 66.7 | 30.8 | 0.115 | 41.7 | 23.1 | 0.411 | 0.632 | 0.125 | 0.375 |
| **Insomnia** | 58.3 | 46.2 | 0.695 | 50.0 | 23.1 | 0.226 | 50.0 | 38.5 | 0.695 | 0.640 | 1.000 | 1.000 |
| **Vomiting** | 41.7 | 46.2 | 1.000 | 33.3 | 15.4 | 0.378 | 41.7 | 15..4 | 0.202 | 0.142 | 1.000 | 0.125 |
| **Nervousness** | 75.0 | 46.2 | 0.226 | 58.3 | 46.2 | 0.695 | 58.3 | 38.5 | 0.434 | 0.183 | 0.727 | 1.000 |
| **Aggressions** | 41.7 | 23.1 | 0.411 | 50.0 | 7.7 | **0.030** | 33.3 | 15.4 | 0.378 | 0.480 | 1.000 | 1.000 |
| **Anxiety** | 41.7 | 23.1 | 0.411 | 8.3 | 15.4 | 1.000 | 25.0 | 30.8 | 1.000 | 0.481 | 0.625 | 1.000 |
| **Depressions** | 50.0 | 38.5 | 0.695 | 16.7 | 7.7 | 0.593 | 50.0 | 15.4 | 0.097 | 0.090 | 1.000 | 0.250 |
| **Libido disturbance** | 50.0 | 38.5 | 0.695 | 25.0 | 15.4 | 0.645 | 41.7 | 30.8 | 0.688 | 0.752 | 1.000 | 1.000 |
| **Potence disturbance** | 16.7 | 15.4 | 1.000 | 8.3 | 7.7 | 1.000 | 16.7 | 7.7 | 0.593 | 0.485 | 1.000 | 1.000 |
| **Poor appetite** | 66.7 | 23.1 | **0.047** | 58.3 | 7.7 | **0.011** | 33.3 | 23.1 | 0.673 | 0.814 | 0.219 | 1.000 |
| **Constipation** | 41.7 | 23.1 | 0.411 | 33.3 | 7.7 | 0.160 | 33.3 | 38.5 | 1.000 | 0.329 | 1.000 | 0.500 |
| **Itching** | 16.7 | 30.8 | 0.645 | 8.3 | 23.1 | 0.593 | 16.7 | 30.8 | 0.645 | 0.656 | 1.000 | 1.000 |
| **Feelings of coldness** | 75.0 | 30.8 | **0.047** | 58.3 | 15.4 | **0.041** | 41.7 | 23.1 | 0.411 | 0.250 | 0.219 | 1.000 |
| **Heart pounding** | 33.3 | 30.8 | 1.000 | 25.0 | 7.7 | 0.322 | 8.3 | 30.8 | 0.322 | 0.157 | 0.375 | 1.000 |
| **Muscular tension** | 66.7 | 23.1 | **0.047** | 41.7 | 15.4 | 0.202 | 25.0 | 15.4 | 0.645 | 0.781 | 0.125 | 1.000 |
| **Yawning** | 58.3 | 30.8 | 0.238 | 58.3 | 15.4 | **0.041** | 33.3 | 15.4 | 0.378 | 0.283 | 0.625 | 0.375 |
| **General aches and pains** | 41.7 | 30.8 | 0.688 | 33.3 | 15.4 | 0.378 | 25.0 | 7.7 | 0.322 | 0.819 | 0.250 | 0.500 |
| **Total Score of Somatic complaints (range 0-10)** | 4.9 (1.8) | 3.3 (2.2) | 0.077 | 3.8 (2.1) | 1.6 (1.6) | **0.011** | 3.7 (2.4) | 2.5 (2.5) | 0.270 | 0.068 | 0.201 | 0.194 |

QOL scores for the study completers after 6 months of treatment are also shown in Table 2. Patients retained in the buprenorphine or methadone program showed significant differences only within the overall satisfaction score ($p$ = 0.030), in which the patients of the buprenorphine group showed the more favorable values.

The outcome measures for physical symptoms after 6 months of treatment are shown in Table 2. Buprenorphine showed less physical symptoms regarding stomach cramps ($p$ = 0.041), fatigue or tiredness ($p$ = 0.011), aggressions ($p$ = 0.030), poor appetite ($p$ = 0.011), feelings of coldness ($p$ = 0.041), and yawning ($p$ = 0.041).

At the end of the investigation period (3 years), the buprenorphine-maintained group showed significantly higher QOL scores within leisure time ($p$ = 0.035), housing ($p$ = 0.010), and law and security ($p$ = 0.035) than methadone participants (Table 2). The outcome measures for physical symptoms demonstrate significant advantages for buprenorphine regarding stomach cramps ($p$ = 0.030) and fatigue or tiredness ($p$ = 0.047).

Analysis of covariance adjusting quality of life scores at 36 months for baseline values did not show significant differences between the two substitution groups (Table 2). Logistic regression to adjust nominal data sets showed less physical symptoms within the buprenorphine group regarding stomach cramps ($p = 0.037$) and fatigue and tiredness ($p = 0.034$).

After 3 years, patients who received buprenorphine treatment showed a statistically significant increase of the QOL score regarding law and security ($p = 0.026$). No statistically significant differences of the QOL score occurred for patients who received methadone treatment. Regarding physical symptoms, the methadone and buprenorphine participants showed no significant changes (Table 2).

A total number of 910 urine screening tests were carried out randomly to detect additional consumption. The urine samples were counted as positive if at least one positive urine test was found within the last 4 weeks. The completer groups showed, throughout the first 2 months, no significant differences in terms of illicit cocaine, opioids, amphetamine, or benzodiazepine use (Table 3). The outcome measures for positive urine samples after 6 months of treatment are shown in Table 3. Thereby, the buprenorphine group showed less additional consumption of opioids ($p = 0.041$). Table 3 further demonstrates differences after 3 years of maintenance treatment in terms of illicit cocaine, amphetamine, and opioid use observed within both of the substitution groups, in which the patients of the buprenorphine group showed the more favorable values. After 3 years of substitution treatment, the buprenorphine-maintained group showed significantly less additional consumption of benzodiazepines ($p = 0.015$) compared with the methadone participants (Table 3).

Within the buprenorphine and methadone group, no significant changes were observed regarding additional consumption.

**TABLE 3**
**Percentages of Positive Urine Samples Throughout a Study Period of 3 Years (2001–2004): Completer and Noncompleter Analysis**

| positive urine samples completers | Methadone at 2 months [%] | Buprenorphine at 2 months [%] | p-value 2 months | Methadone at 6 months [%] | Buprenorphine at 6 months [%] | p-value 6 months | Methadone after 3 years [%] | Buprenorphine after 3 years [%] | p-value 3 years | p-value methadone group between baseline and 3 years | p-value buprenorphine group between baseline and 3 years |
|---|---|---|---|---|---|---|---|---|---|---|---|
| amphetamine | 0.0 | 0.0 | - | 0.0 | 0.0 | - | 16.7 | 7.7 | 0.593 | 0.500 | 1.000 |
| benzodiazepine | 33.3 | 46.2 | 0.688 | 41.7 | 30.8 | 0.688 | 66.7 | 15.4 | 0.015 | 0.219 | 0.125 |
| cocaine | 25.0 | 15.4 | 0.645 | 25.0 | 7.7 | 0.322 | 33.3 | 7.7 | 0.160 | 1.000 | 1.000 |
| opioids | 50.0 | 15.4 | 0.097 | 58.3 | 15.4 | 0.041 | 50.0 | 23.1 | 0.224 | 1.000 | 1.000 |

| positive urine samples non-completers | Methadone at 2 months [%] | Buprenorphine at 2 months [%] | p-value 2 months | Methadone at 6 months [%] | Buprenorphine at 6 months [%] | p-value 6 months |
|---|---|---|---|---|---|---|
| amphetamine | 0.0 | 6.3 | 1.000 | 0.0 | 6.3 | 1.000 |
| benzodiazepine | 50.0 | 68.8 | 0.441 | 33.3 | 50.0 | 0.459 |
| cocaine | 16.7 | 12.5 | 1.000 | 16.7 | 6.3 | 0.560 |
| opioids | 66.7 | 37.5 | 0.252 | 66.7 | 25.0 | 0.053 |

### Noncompleter Analysis

QOL characteristics and physical symptoms of the noncompleter groups are detailed in Table 4. According to the self-reported Lancashire Quality of Life Profile, no statistically significant differences at baseline were found. QOL scores for noncompleters after 6 months were not statistically different. Regarding physical symptoms, no significant changes between the two groups were observed. The total score of physical symptoms showed significant improvements within the buprenorphine ($p = 0.016$) and within the methadone group ($p = 0.043$). At 2 and 6 months of substitution treatment, the buprenorphine-maintained group and methadone participants showed no significant differences regarding additional consumption (Table 3).

**TABLE 4**
**Subjective Scores of the Lancashire Quality of Life Profile and Percentage of Physical Symptoms: Noncompleter Analysis**

| Satisfaction Scores of Different Domains[1] (Range 1–7) | Meth. Baseline | Bupr. Baseline | *p*-Value Baseline | Meth. at 6 Months | Bupr. at 6 Months | *p*-Value at 6 Months |
|---|---|---|---|---|---|---|
| Job | 4.2 (2.1) | 3.6 (2.0) | 0.507 | 4.8 (1.6) | 4.0 (1.7) | 0.241 |
| Leisure time | 4.1 (1.0) | 3.9 (1.5) | 0.732 | 5.2 (1.1) | 4.5 (1.3) | 0.110 |
| Finances | 2.5 (1.5) | 3.0 (2.0) | 0.664 | 3.7 (1.8) | 3.9 (1.8) | 0.664 |
| Housing | 4.3 (1.6) | 5.0 (1.5) | 0.205 | 5.1 (1.6) | 5.1 (1.6) | 0.982 |
| Law and security | 5.3 (1.3) | 4.8 (1.6) | 0.423 | 5.8 (0.7) | 5.2 (1.5) | 0.371 |
| Family | 4.7 (1.6) | 4.1 (2.5) | 0.698 | 5.0 (1.2) | 4.1 (2.3) | 0.397 |
| Partnership | 4.2 (2.2) | 4.6 (2.1) | 0.698 | 4.9 (1.8) | 4.9 (1.9) | 0.945 |
| Friends and acquaintances | 4.9 (1.4) | 5.0 (1.4) | 0.982 | 5.4 (1.2) | 5.0 (1.6) | 0.599 |
| Physical health | 4.0 (1.9) | 3.6 (1.6) | 0.599 | 5.1 (1.4) | 4.6 (1.5) | 0.371 |
| Psychical health | 3.3 (1.7) | 3.8 (1.7) | 0.599 | 4.7 (1.4) | 4.0 (1.8) | 0.324 |
| Self-esteem[2] | 7.0 (2.2) | 5.2 (3.0) | 0.100 | 7.5 (1.9) | 6.9 (2.9) | 0.837 |
| Overall satisfaction | 3.2 (1.6) | 3.6 (1.4) | 0.507 | 4.3 (0.9) | 4.4 (1.3) | 0.909 |
| **Percentage of Physical Symptoms (%)** | | | | | | |
| Stomach cramps | 33.3 | 50.0 | 0.459 | 25.0 | 6.3 | 0.285 |
| Twitching of muscles | 33.3 | 68.8 | 0.125 | 33.3 | 50.0 | 0.459 |
| Fatigue or tiredness | 58,3 | 56.3 | 1.000 | 50.0 | 50.0 | 1.000 |
| Runny eyes | 41.7 | 50.0 | 0.718 | 16.7 | 25.0 | 0.673 |
| Inability to fall asleep | 41.7 | 68.8 | 0.250 | 58.3 | 56.3 | 1.000 |
| Insomnia | 58.3 | 62.5 | 1.000 | 58.3 | 56.3 | 1.000 |
| Vomiting | 33.3 | 37.5 | 1.000 | 25.0 | 31.3 | 1.000 |
| Nervousness | 75.0 | 81.3 | 1.000 | 83.3 | 68.8 | 0.662 |
| Aggressions | 41.7 | 56.3 | 0.704 | 41.7 | 31.3 | 0.698 |
| Anxiety | 50.0 | 56.3 | 1.000 | 50.0 | 50.0 | 1.000 |
| Depressions | 50.0 | 62.5 | 0.702 | 58.3 | 56.3 | 1.000 |
| Libido disturbance | 58.3 | 56.3 | 1.000 | 41.7 | 43.8 | 1.000 |
| Potency disturbance | 25.0 | 18.8 | 1.000 | 25.0 | 25.0 | 1.000 |
| Poor appetite | 41.7 | 62.5 | 0.445 | 33.3 | 50.0 | 0.459 |
| Constipation | 50.0 | 43.8 | 1.000 | 8.3 | 31.3 | 0.196 |
| Itching | 41.7 | 31.3 | 0.698 | 8.3 | 31.3 | 0.196 |
| Feelings of coldness | 75.0 | 62.5 | 0.687 | 50.0 | 31.3 | 0.441 |
| Heart pounding | 41.7 | 56.3 | 0.704 | 33.3 | 12.5 | 0.354 |
| Muscular tension | 50.0 | 56.3 | 1.000 | 16.7 | 43.8 | 0.223 |
| Yawning | 33.3 | 37.5 | 1.000 | 16.7 | 43.8 | 0.223 |
| General aches and pains | 50.0 | 62.5 | 0.702 | 25.0 | 25.0 | 1.000 |
| Total score of somatic complaints (range 0–10)[3] | 4.7 (2.5)[1] | 5.4 (2.1) | 0.478 | 3.6 (2.5) | 3.9 (2.1) | 0.837 |

1 Mean (standard deviation)
2 Satisfaction scores range 1–10.
3 Somatic complaints scores range 0–10 (0 means none of the 21 somatic complaints, 10 means all of the 21 somatic complaints).

#### Comparison of Completers and Noncompleters

Regarding baseline values of the total sample (completers and noncompleters), QOL and somatic complaints did not show significant differences between the two treatment groups.

At baseline, the completer group showed the more favorable values regarding physical symptoms ($p = 0.020$). The methadone completer and noncompleter participants did not show any significant differences regarding QOL characteristics or physical symptoms.

QOL characteristics at 6 months of treatment of the buprenorphine completer and noncompleter groups differed significantly regarding job ($p = 0.013$), family, and total score of physical symptoms ($p = 0.002$), in which the completer group showed the more favorable values.

### DISCUSSION

The application of patient-centered health outcomes to medicine represents an important advance in the humanistic delivery of health care[74,75]. Therefore, QOL has become an important endpoint in clinical trials and studies of medical interventions for many chronic diseases, but has not been widely studied in the substance abuse field[7,13,14,15,16,17,18,19,20]. QOL measures are usually better than more traditional, clinical measures for evaluating the social and emotional outcomes of treatments and disease processes, and giving an overall picture of how treatments are affecting the patient's ability to function in life[18,74,76,77,78,79,80].

The present study is one of the first reports presenting a follow-up, aimed at comparing oral methadone with sublingual buprenorphine, as measured by QOL, physical symptoms, and additional consumption after a treatment period of 3 years (2001–2004), obtained from patients retained in one of the two maintenance programs.

Before discussing our results, we would like to point out some limitations of our study. Due to the open nature of the study and the fairly low number of subjects, results should be regarded with caution. The present study has a naturalistic design. As mentioned, history and physical examination supported the judgment on the part of the physician that the patient was a candidate for methadone or sublingual buprenorphine maintenance program and that such treatment was indicated on the basis of thorough standard clinical evaluations[67,68,69,70,71,72,73]. There was no random assignment to one of the two treatment groups. This study, therefore, did not address whether there might be a selection effect for addiction treatment. Even if the patient self-selection worked and arrived at a group of patients, there is no evidence that the majority of those who were "eligible" for one of the maintenance groups got any clinical benefit from it. The use of a standardized instrument showing improvements in QOL is an important strength of the study. Other QOL instruments have rarely been applied in this context[7,16,17,18]. The use of a standardized forensic urinalysis is another important strength of the study.

Our results showed nearly the same retention rate of 50% for methadone and 45% for sublingual buprenorphine after a treatment period of 3 years. These retention rates may be considered rather successful. Fischer et al.[39] reported retention rates of 71% for methadone and 38% for buprenorphine within a much shorter study period of 24 weeks. During a 16-week period, Johnson et al.[52] reported retention rates of 32% for methadone and 42% for buprenorphine. However, most of these studies examined the impact of treatment under strict and closely supervised conditions that do not reflect the various ways in which these substitution drugs are used once they are made available routinely[81].

Calculated dose conversion for equivalent doses in both programs did not show differences throughout the study[82]. Therefore, obtained completer results may not be regarded as a reflection of dose effect. At the end of the study period, the mean methadone dose was 55.4 mg and the mean buprenorphine dose was 8.1 mg. This result confirms the findings of Strain et al.[36], Uehlinger et al.[50], and Pani et al.[54]. These randomized, controlled studies of up to 6-months duration generally demonstrated comparable efficacy with buprenorphine 8–12 mg/day and methadone 30–90 mg/day in promoting retention in treatment and reducing illicit opioid use. One randomized, double-blind, dose-ranging trial was set up to compare the efficacy of buprenorphine 8 mg/day with 1 mg/day for the treatment of opioid dependence[38]. Data were also collected on 4- and 16-mg daily doses, although these were considered as secondary outcomes. At 16 weeks, the 1-mg group had significantly poorer retention (40%) than both the 8-mg group (52.1%) and the 16-mg group (60.8%). Urine test results in this study demonstrated less use of illicit opioids in patients assigned the 8-mg dose compared to the 1-mg dose. There were no significant differences between the 8- and 16-mg group in any endpoint.

However, our result does not confirm the findings of Barnett et al.[41]. Barnett et al. reported that subjects receiving a daily dose between 8 and 12 mg of buprenorphine were 1.26 times more likely to drop out of treatment than subjects receiving 50–80 mg/day of methadone, and that they were 8.3% more likely to have a positive urinalysis for opioids. Regarding our results, we agree with Barnett et al. that an 8- to 12-mg/day dose of buprenorphine is more effective than low-dose methadone. Barnett et al. suggested that a modest change in buprenorphine dose may result in substantially different outcomes. Leavitt et al.[82,83] reported that the successful treatment of some patients requires doses well in excess of 100 mg/day methadone. However, dose finding was not likely to have a major impact on the results of our study, as the treating physician had sufficient experience in maintenance treatment with both substances. Additional studies will need to determine if buprenorphine doses of higher than 12 mg/day are more effective. Davids and Gastpar[84] suggest in their recent review that buprenorphine in a dosage range 8–16 mg/day guarantees an effective maintenance therapy of opioid-addicted patients. The variation between different trials may be due to differences in dose levels, patient exclusion criteria, and provision of psychosocial treatment[39].

When the subset of methadone completers assessed at baseline was compared with the buprenorphine subjects, there were only a few significant differences regarding QOL scores, in which patients of the buprenorphine program showed the more-favorable values. At the end of the study, there were no significant differences regarding QOL scores between the two groups.

Opioid addicts within a methadone or buprenorphine maintenance program for a 6-month period experienced relevant improvements in QOL. After a 3-year period, addicts in both of the maintenance programs experienced less-favorable QOL scores compared with a treatment time of 6 months.

Habrat et al.[85] found that patients before admission to a methadone program had an extremely low QOL. After 6-months participation, QOL increased significantly in seven from eight subscales of the SF-36. During the next 6 months, QOL unexpectedly decreased without clear reasons, but not to prior level. Habrat et al. found no significant differences in QOL between men and women, although men benefitted from the methadone program more than women.

Regarding our QOL results, successful buprenorphine or methadone responders cannot easily be distinguished. QOL characteristics and physical symptoms of the noncompleter groups showed no statistically significant differences at baseline and after 6 months of treatment. Also regarding physical symptoms, no significant differences between the two groups were observed.

The meta-analysis of Barnett et al.[41] enrolled different types of patients and showed that buprenorphine was most successful, involving only few psychosocial interventions. Patients at our department received the same level of care in the different treatment groups. Office visits regarding psychosocial care were, therefore, at least every 4 weeks for all patients. Regarding psychosocial support and treatment outcome, Gerra et al.[86] found that the retention rate seems to be influenced by dose, psychosocial functioning, and not by psychiatric comorbidity in methadone maintenance patients. Gerra et al. found no relationship between retention rate and dose, or retention rate and psychosocial functioning, evidenced for buprenorphine patients. As evidenced for retention, substance abuse history and psychosocial functioning, in this study, appeared unable to influence urinalyses results in buprenorphine patients. Alternatively, psychiatric comorbidity was found unrelated to urinalyses results in methadone patients. Gerra et al. concluded that psychosocial functioning and addiction severity cannot be used as valuable predictors of buprenorphine treatment outcome.

In an attempt to characterize treatment “nonresponsive” patients and to identify their specific treatment needs, Belding et al.[87] compared them to a group of patients with comparable treatment tenures, but whose urinalysis results indicated little or no recent illicit opiate use. Contrary to expectations, opioid use itself was virtually the only feature that clearly distinguished the two groups. “Responders” and “nonresponders” did not differ significantly on measures of psychosocial problem severity in any other area, nor did they differ in their treatment service utilization[87].

Although many studies document the use of the maintenance treatment to classify opioid users, the predictive validity of subgroups who respond well to psychosocial support is rarely reported[89]. However, study results suggest the relation between psychosocial problems and treatment outcome may be more complex than is commonly assumed[86,87,88,89,90,91].

Further investigations and studies should be planned to identify possible characteristics of subgroups that respond well to buprenorphine or methadone treatment. It is important to continue to compare the benefits of both types of treatment, taking other factors into account, such as physical health, mental health, psychosocial care, and social functioning.

Effectiveness may depend on other treatment factors. Ventegodt et al.[16] found that the group with the lowest QOL was found to be persons who had used heroin, morphine, methadone, and a mixture of alcohol and tranquilizers (10–20% below the group with the highest QOL). Observational studies conducted in France since 1996 indicate an overall improvement in the living conditions of users undergoing a buprenorphine treatment[9,92,93], especially with respect to social housing, employment, drug (heroin and cocaine) use, and injection behavior[94,95].

Patients on maintenance treatment are expected to use drugs (especially heroin) less frequently, stabilize or improve their social situation, enjoy greater access to medical care, and to commit fewer criminal offences[81]. At the end of the investigation period of 3 years in this study, the buprenorphine-maintained group showed less additional amphetamine, opioid, cocaine, and benzodizepine consumption than the methadone group. A significant difference was observed in additional consumption of benzodiazepines between the two groups in the current study, confirming the findings of Fischer et al.[39], Eder et al.[96], and Schottenfeld et al.[97]. In opposition to our results, Thirion et al.[98] found a high level of benzodiazepine consumption (43%). The significant decrease of additional consumption of benzodiazepines (only 15%) in the completer group maintained on buprenorphine could represent a subgroup of patients who respond very well to this opioid. Benzodiazepines are often taken for recreational purposes by an increasing number of drug abusers. They commonly form part of a polysubstance abuse pattern[99]. Benzodiazepines are mainly taken along with the primary drug, but are sometimes also used alone as an alternative or in times of shortage[99,100].

Schottenfeld et al.[97] found some gender differences regarding additional consumption of opioids. The decrease in the number of positive urinalyses confirms an advantage over methadone and the clinical usefulness of buprenorphine, as shown in other studies[36,50,54]. Guichard et al.[81] suggest that drug injection within a buprenophine treatment population is particularly related to social conditions (unstable housing) and a higher daily dosage.

In the methadone and buprenorphine treatment group, a total of 13 patients discontinued treatment for change of substitution treatment. The desire of participants to change substitution treatment usually has specific reasons. Nowadays, many clients in Austria tend to get a slow-release oral morphine substitution[101,102,103]. Since the introduction of slow-release oral morphine for opioid substitution in 1999, most of the opioid clients in Austria tend to develop a morphine addiction[104]. The opioid-dependent individual in Austria, as referred here, is rather physically dependent on morphine (slow-release oral morphine) than on heroin. The Austrian situation is, therefore, unique compared with other European countries. The abuse of slow-release oral morphine by opioid-dependent individuals is a “street phenomenon” that has received clinical attention in Austria[105]. The increase in the use of morphines over the period 2000–2003 occurred in Austria about 6 months after their introduction in 1999 as a maintenance substance for opioid addiction. Due to a continuing increase in the amount of “black-market morphine” prescribed and nonprescribed, morphine deaths in Austria have increased since 2000[104], while total drug-related deaths did not increase any more. In 2002, there were 101 deaths (of a total 139) recorded in Austria that were attributable to morphine abuse. Heroin was detected in only 21 deaths[104]. In a Viennese study of a needle and syringe exchange program, Haltmayer et al.[106] found traces of morphine in 37% and heroin only in 17%. Despite these changes regarding the abuse of morphine, outcomes have rarely been studied systematically[15,103,107,108]. Morphine, typically injected with a syringe, has many side effects. The most dangerous one is respiratory depression. The fact remains, however, that intravenous oral morphine abuse has clear implications for infections, inflammations, and other severe side effects[109,110,111].

The pharmacological properties of buprenorphine suggest that it should be safer and result in less physical dependence than methadone[35,36,37,38,39,45,46,47,48]. With increasing use of opioids for treatment of pain and the availability of sublingual buprenorphine in the U.S. for treatment of opioid dependence, clinical assessment of opiate withdrawal intensity has received renewed interest. Buprenorphine, a partial opiate agonist at the mu receptor, can precipitate opiate withdrawal in patients with a high level of opioid dependence who are not experiencing opioid withdrawal[112]. Health status at entry to the buprenorphine or methadone maintenance treatment was equal. Opioid addicts improved their QOL and health status when treated with methadone or buprenorphine. Regarding long-term outcomes, buprenorphine treatment showed moderately better results than the treatment with methadone. This result confirms the findings of Liu et al.[66]. Liu et al. suggest that the withdrawal of buprenorphine produces a symptom/sign that is qualitatively similar to that of opiate agonist, but the intensity is considerably less. In conclusion, the results of Liu et al. suggest that buprenorphine produces low, drug-dependent potential because of its unique pharmacological properties. The study of Liu et al. supports the postulate that buprenorphine as a partial agonist at the mu-opioid receptor, unlike morphine and methadone that are all full agonists at this receptor, has a unique pharmacological profile and a significantly lower drug-dependent potential (both physical and psychic) than morphine and methadone. Liu et al. conclude that physical and psychic dependence of buprenorphine can be regarded as low.

### CONCLUSIONS

Heroin addiction is a chronic, relapsing disease that is difficult to cure, but stabilization and harm reduction can greatly increase the life expectancy and the QOL of the patient, his direct environment, and society as a whole[17]. Although it is very tempting to link participation in a buprenorphine maintenance program to improved health and QOL scores, other factors may also contribute to the improved health and QOL scores of patients[9,16,92,93]. When considering the amount of opioid and cocaine consumed, it appears that patients who participated in the buprenorphine program were less “heavy” heroin users than those who followed the methadone treatment. However, regarding our urine results, we consider that all patients were “heavy” opioid users. Despite the considerable number of trials that have been carried out on this topic, they are very heterogeneous as far as the comparisons and outcomes are considered. This prevented many of them from being incorporated into a quantitative meta-analysis[34,40,41]. Consensus in measurements and results should be reached among researchers involved in the evaluation of the effectiveness of treatments for opioid addiction in order to produce consistent outcomes in the measuring and reporting of results from clinical trials[113].

In summary, the present data indicate that buprenorphine is a useful long-term alternative for maintenance treatment of opioid-dependent patients. However, larger studies should identify who would benefit more from buprenorphine or methadone maintenance treatment. Future trials should consider the effect of dose, treatment setting, and personal characteristics of patients. We conclude, in agreement with Smith and Larson[18], that QOL provides information about functioning and well being that is not captured by traditional measures of substance use, and may soon begin to play a more prominent role in evaluating the effectiveness of treatment services for substance abusers.

### ACKNOWLEDGMENTS

We would like again to express our appreciation to all the people we interviewed for their time and energy.

### BIOSKETCHES

**Salvatore M. Giacomuzzi**, **PhD.** Dr. Giacomuzzi's areas of interest include addiction research, evaluation for different treatment programs with regard to quality of life assessment; driving capacity and maintenance treatment; psychotherapy and addiction; addictions/adolescents and children; resocialization programs; resocialization and addiction; addiction and medical imaging regarding effects of psychotropic substances. University Department of Psychiatry, Anichstraße 35, 6020 Innsbruck, Austria. Tel: 0043-512-504-24750; Fax: 0043-512-504-24764. E-mail: salvatore.giacomuzzi@uibk.ac.at

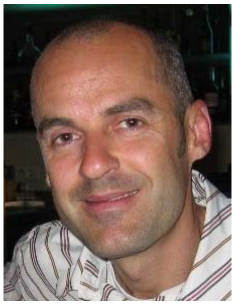

**Markus Ertl**, **PhD.** Dr. Ertl's areas of interest include psychological testing; children's behavior difficulties; addiction research; evaluation for different treatment programs with regard to quality of life assessment; driving capacity and maintenance treatment; addictions/adolescents and children; resocialization programs; resocialization and addiction. University Department of Psychiatry, Anichstraße 35, 6020 Innsbruck, Austria. Tel: 0043-512-504-81446; Fax: 0043-512-504-24764. E-mail: markus.ertl@uibk.ac.at

**Georg Kemmler**, **PhD.** is a biostatistician. His areas of interest include statistical and psychometrical issues in psychiatry, psychooncology, and quality of life research. University Department of Psychiatry, Anichstraße 35, 6020 Innsbruck, Austria. Tel: 0043-512-504-28636; E-mail: georg.kemmler@uibk.ac.at

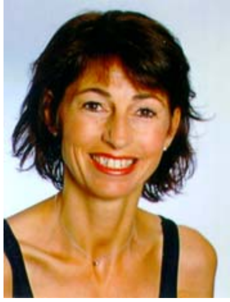

**Yvonne Riemer**, **MD.** Dr. Riemer's areas of interest include addiction treatment; psychiatric disorders and addiction treatment; addiction treatment and adolescents; Hep C and optimization of maintenance treatment; HIV and addiction treatment; quality of Life study. University Department of Psychiatry, Anichstraße 35, 6020 Innsbruck, Austria. Tel: 0043-512-504-81446; Fax: 0043-512-504-24764. E-mail: yvonne.riemer@uibk.ac.at

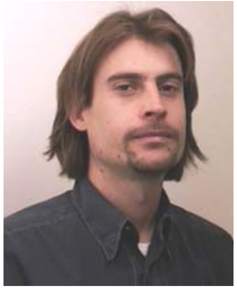

**Alexander Vigl**, **PhD.** Dr. Vigl's areas of interest include children's behavior difficulties; addiction research; driving capacity and maintenance treatment; addictions/adolescents and children; resocialization programs; resocialization and addiction. University Department of Psychiatry, Anichstraße 35, 6020 Innsbruck, Austria. Tel: 0043-512-504-81446; Fax: 0043-512-504-24764. E-mail: alexander.vigl@uklibk.ac.at
